# Verteporfin inhibits growth of human glioma *in vitro* without light activation

**DOI:** 10.1038/s41598-017-07632-8

**Published:** 2017-08-08

**Authors:** Ahmad Al-Moujahed, Katarzyna Brodowska, Tomasz P. Stryjewski, Nikolaos E. Efstathiou, Ioannis Vasilikos, Joanna Cichy, Joan W. Miller, Evangelos Gragoudas, Demetrios G. Vavvas

**Affiliations:** 1 Retina Service, Angiogenesis Laboratory, Massachusetts Eye and Ear Infirmary, Department of Ophthalmology, Harvard Medical School, Boston, Massachusetts 02114 USA; 2 University Medical Center Freiburg, Freiburg, Germany; 3 Department of Immunology, Faculty of Biochemistry, Biophysics and Biotechnology, Jagiellonian University, Krakow, Poland

## Abstract

Verteporfin (VP), a light-activated drug used in photodynamic therapy for the treatment of choroidal neovascular membranes, has also been shown to be an effective inhibitor of malignant cells. Recently, studies have demonstrated that, even without photo-activation, VP may still inhibit certain tumor cell lines, including ovarian cancer, hepatocarcinoma and retinoblastoma, through the inhibition of the YAP-TEAD complex. In this study, we examined the effects of VP without light activation on human glioma cell lines (LN229 and SNB19). Through western blot analysis, we identified that human glioma cells that were exposed to VP without light activation demonstrated a downregulation of YAP-TEAD-associated downstream signaling molecules, including c-myc, axl, CTGF, cyr61 and survivin and upregulation of the tumor growth inhibitor molecule p38 MAPK. In addition, we observed that expression of VEGFA and the pluripotent marker Oct-4 were also decreased. Verteporfin did not alter the Akt survival pathway or the mTor pathway but there was a modest increase in LC3-IIB, a marker of autophagosome biogenesis. This study suggests that verteporfin should be further explored as an adjuvant therapy for the treatment of glioblastoma.

## Introduction

Gliomas are the most common primary brain tumors^1, 2^. Histopathologically, they are classified by the World Health Organization (WHO) as astrocytoma, oligodendroglioma, mixed oligoastrocytoma, and ependymoma with glioblastoma (WHO astrocytoma grade IV) being the most common adult glioma^1, 2^. In the eye, optic nerve gliomas (ONGs) comprise 2–5% of all pediatric central nervous system (CNS) tumors and are the most common CNS tumors in patients with neurofibromatosis type 1 (NF1)^3^.

Signs and symptoms of gliomas depend on the site of CNS that is affected. For instance, brain gliomas cause headache, vomiting, and seizures^1^, while the presenting clinical signs of ONGs include mild to profound vision loss, proptosis, optic disk swelling or pallor, ophthalmoplegia, and strabismus^4–8^. Gliomas are rarely curable and the prognosis of patients with high grade gliomas is usually poor^1, 2^. Neither surgery, chemotherapy, nor radiation has been demonstrated to prolong survival in cases of malignant optic nerve glioma^4–8^. Moreover, even in cases of low grade glioma in children, treatment is associated with significant morbidity, with risks including iatrogenic damage to the optic nerve, cortical atrophy, degenerative vascular changes and increased incidence of secondary tumors^9^.

A recent experimental innovation in cancer therapy has been the use of porphyrins, which are organic heterocyclic molecules consisting of four modified pyrrole units interconnected via methane bridges^10^. A unique feature of porphyrins is their ability to act as photosensitizers^10^. One clinical application of porphyrins in the eye is through photodynamic therapy (PDT), a Food and Drug Administration (FDA)-approved intervention to treat discrete subfoveal choroidal neovascular membranes secondary to age related macular degeneration^11^. PDT involves intravenously administering verteporfin (trade name Visudyne), which accumulates in neovascular subretinal vessels, and is then activated upon exposure to 693 nm, low-energy, nonthermal infrared laser. Activation of verteporfin (VP) produces free radicals in the abnormal vessels resulting in their destruction through multiple mechanisms including a direct cytotoxic effect, promotion of vascular thrombosis, as well as an immune mediated effect. The highly localized effect spares the overlying fovea and retinal pigment epithelium. Since its first application, its use has expanded worldwide and has preserved vision in tens of thousands of patients^12^.

More recently, PDT has also been experimentally used as a light-based therapeutic modality for several human malignancies^13–17^. It has been proposed that the therapeutic mechanism of treating malignancies with VP is not only through its light-activated destruction of neovascular vessels, but also as a possible inducer of apoptosis or autophagy in malignant cells^18^.

Yes Associated Protein (YAP), a candidate oncogene on the human chromosome 11q22 amplicon, and component of the Hippo-pathway has been associated with human tumorigenesis^19–21^. In addition, high YAP nuclear levels are present^22–24^ and have been linked to chemoresistance^25–28^ in various cancer types. YAP works as a transcriptional coactivator and binds to several DNA-binding transcription factors; the best characterized have been the TEAD family of transcription factors^29^. Recent studies have shown that VP may disrupt the YAP-TEAD complex and inhibit growth of hepatocellular carcinoma and ovarian cancer without light activation^10, 30^. In addition, we recently demonstrated verteporfin’s ability to interfere with the YAP-TEAD signaling pathway, resulting in a downregulation of proto-oncogenes, molecules involved in angionesesis and cell migration, and a reduction in the pluripotency cell marker Oct-4^31^. Drugs that disrupt YAP-TEAD interaction can have selective effects on malignant cells with minimal toxicity on the surrounding healthy tissues because this pathway is usually not active in normal tissues. This makes these drugs an attractive potential novel therapeutic option. Given the recently characterized role of YAP expression in human glioblastoma^32, 33^ and the need to identify less toxic and more effective therapies for this deadly cancer, we investigated the effects of non-light activated VP on human glioma cells.

## Materials and Methods

### Reagents

Verteporfin (Visudyne) was obtained from Novartis (Novartis, Basel, Switzerland) and MTT (3-(4,5-dimethylthiazol-2-yl)-2,5-diphenyltetrazolium bromide) was purchased from Sigma Aldrich (St.Louis, MO, USA). The following primary antibodies were purchased from Cell Signaling Technology (Danvers, MA, USA) and were diluted 1:1000 unless stated otherwise: c-myc, axl, phospho-S6 ribosomal protein (Ser235/236), phospho-4EBP1 (Thr37/46), LC3B, phospho-p38 MAPK (Thr180/Tyr182), 4-Oct, survivin, pAkt (S473) (1:2000). The following antibodies were purchased from Santa Cruz Biotechnology (Dallas, Texas, USA): cyr61 (1:500), VEGFA (1:500), CTGF (1:500).

### Cell culture

Human glioma cell lines LN229 and SNB19 were purchased from Leibniz Institute DSMZ German Collection of Microorganisms and Cell Lines (Leibniz, Germany) and were grown in DMEM medium (Invitrogen, Grand Island, NY, USA) supplemented with 10% fetal bovine serum (FBS) (ATCC), penicillin (100 μg/ml), streptomycin (100 μg/ml), 10 mM HEPES (all obtained from Invitrogen). Cells were incubated at 37 °C in a humidified atmosphere of 95% air and 5% CO_2_ and split when the cells reached approximately 80% confluence. Cells were continuously protected from light (by the use of aluminum foil) and all experiments were performed in darkness.

### Assessment of growth curves

The cells were seeded in 6-well plates with approximately 100 000 cells per well. One day later, verteporfin was added (final concentrations 2 µg/ml or 10 µg/ml) and cells were incubated for 3 and 6 days. At days 2, 4 and 6, cell number and viability were determined by the trypan blue (0.4%) dye exclusion method. Growth curves were drawn.

### Measurement of cell growth and viability by the MTT (3-(4,5-Dimethylthiazol-2-yl)-2,5-Diphenyltetrazolium Bromide) assay

Cell viability was assessed by the 3-(4,5-dimethylthiazol-2-yl)-2,5-diphenyltetrazolium bromide (MTT) assay. The MTT assay is used to measure the reduction of a tetrazolium compound by the cellular mitochondria, which produces the optically active compound formazan.

Cells were cultured in 96-well plates at an amount of 10 000 cells per well. After 3 and 6 days of treatment with VP, MTT (5 mg/mL in PBS) was added to each well at a 1/10 volume. Cells were incubated for 1 hour at 37 °C and resuspended in DMSO. The absorbance at 595 nm was measured using a microplate reader. Data are displayed as percentage of control.

### Protein extraction and western blot analysis

Cells were incubated for 24 hours in the presence of VP at concentrations of 2 µg/ml and 10 µg/ml. Control cells were treated with PBS. The samples were lysed in M-PER Mammalian Protein Extraction Reagent (Thermo-Scientific, Rockford, IL USA) supplemented with protease (as per manufacturer recommendation; Roche Applied Science) and phosphatase inhibitor cocktails (dilution 1:50; Thermo-Scientific, Pierce Protein Research Products). All cells and samples were continuously protected from light. Ten micrograms of total protein were loaded onto 4–12% Bis-Tris Gel (NuPAGE; Invitrogen). Electrophoresis was performed in darkness using NuPAGE MOPS Running Buffer (Invitrogen) and then samples were transferred onto a PVDF membrane (Millipore, Billerica, MA, USA). After transfer, the membranes were stained with Coomassie blue to ensure equal loading, and then blocked for 45 minutes at room temperature in 5% wt/vol BSA followed by incubation overnight at 4 °C with the above listed rabbit anti-human primary antibodies. Then the membranes were washed three times with 1xTBS 0.1% Tween 20 and incubated for 45 minutes at room temperature with the horseradish peroxidase-labeled secondary anti-rabbit antibody at 1:50000 (Jackson ImmunoResearch, West Grove, PA, USA). The immunoreactive bands were visualized with ECL exposure onto Fuji RX film (Fujifilm,Tokyo, Japan). The results were quantified using ImageJ software (National Institutes of Health, Bethesda, MD; available at http://imagej.nih.gov/ij/) relative to Coomassie blue staining as a loading control.

### Statistical analysis

Data are expressed as mean and standard error of the mean (SEM). Statistical significance was evaluated using the one-way ANOVA test with Dunnett’s modification for multiple means comparison or a t-test for two mean comparisons. A p value of < 0.05 was considered to be significant. Two-tailed tests were used for all comparisons.

## Results

### Verteporfin inhibits cell growth and viability of human glioma cell lines

**Figure 1:**
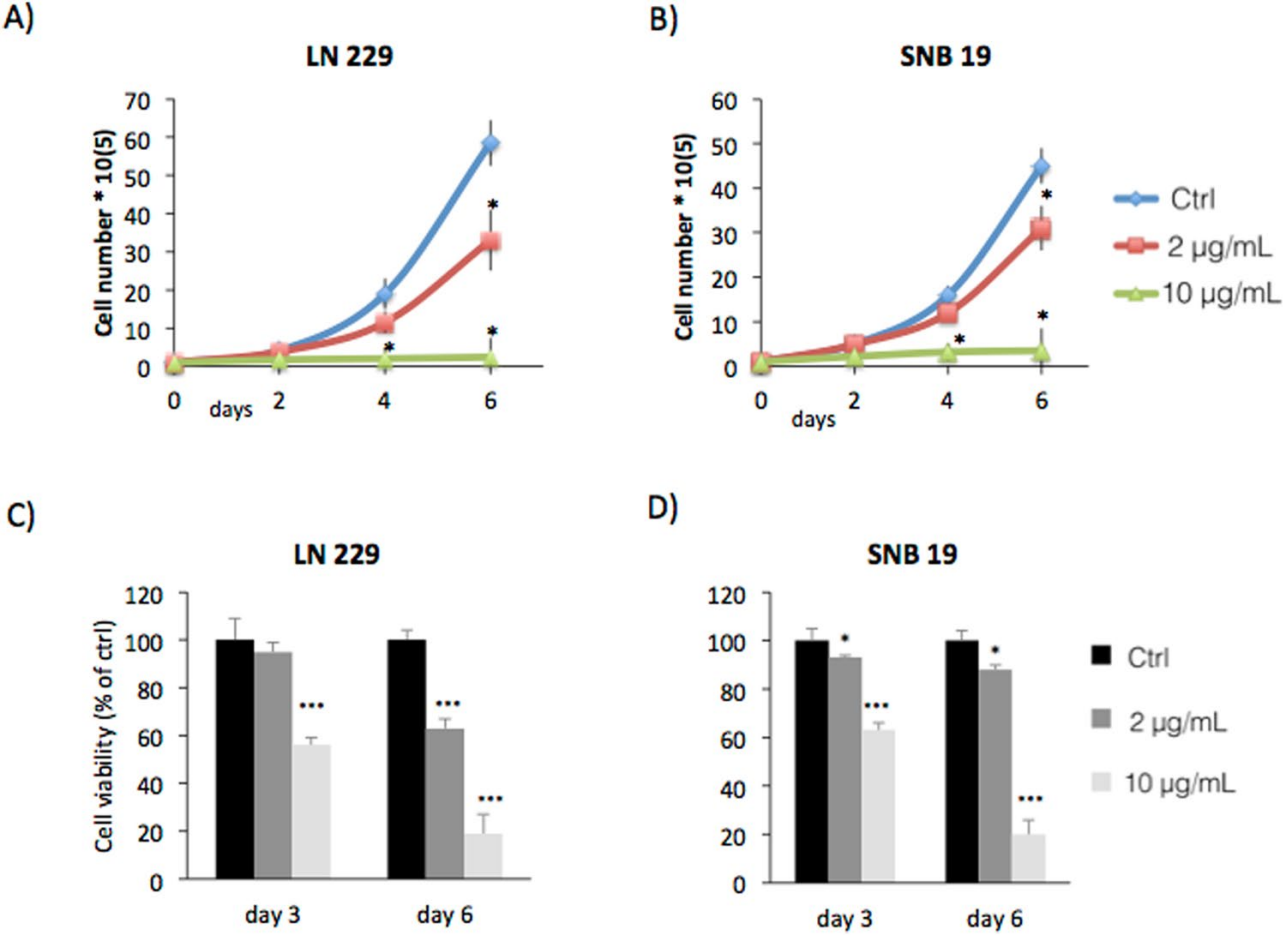
Verteporfin (VP) inhibits growth of glioblastoma LN229 and SNB19 without light activation. Glioblastoma cell lines (SNB19 and LN229) were treated with vehicle (PBS), 2 µg/ml VP or 10 µg/ml of VP for 2,4, and 6 days (**A**,**B**) or 3 and 6 days (**C,D**) protected from light at all steps. (**A**,**B**) Cell growth curves as determined by trypan blue exclusion counting. VP treatment resulted in a dose-dependent inhibition of cell growth. (N = 3, error bars are SE, *p < 0.5 (**C**,**D**) Cell viability assessed by MTT assay. Results expressed as percentage of growth (%) relative to control values. (N = 3, error bars are SE, ***p < 0.001, *p < 0.5).

To determine if VP inhibits human glioma cell growth and proliferation without light activation, we analyzed its effect on the growth of human glioma cell lines LN229 and SNB19. The cell lines were exposed to one of three arms: VP 2 µg/ml, VP 10 µg/ml, or a placebo group with a vehicle control. Cells were continuously protected from light. Both verteporfin exposed cell lines demonstrated dose-dependent decrease in cell growth as measured by trypan blue exclusion testing (Fig. 1A,B). Similarly, a statistically significant inhibition of cell growth and viability was observed in verteporfin treated cells using the MTT assay (Fig. 1C,D).

### VP affects YAP-TEAD signaling pathway in human glioma cells

**Figure 2:**
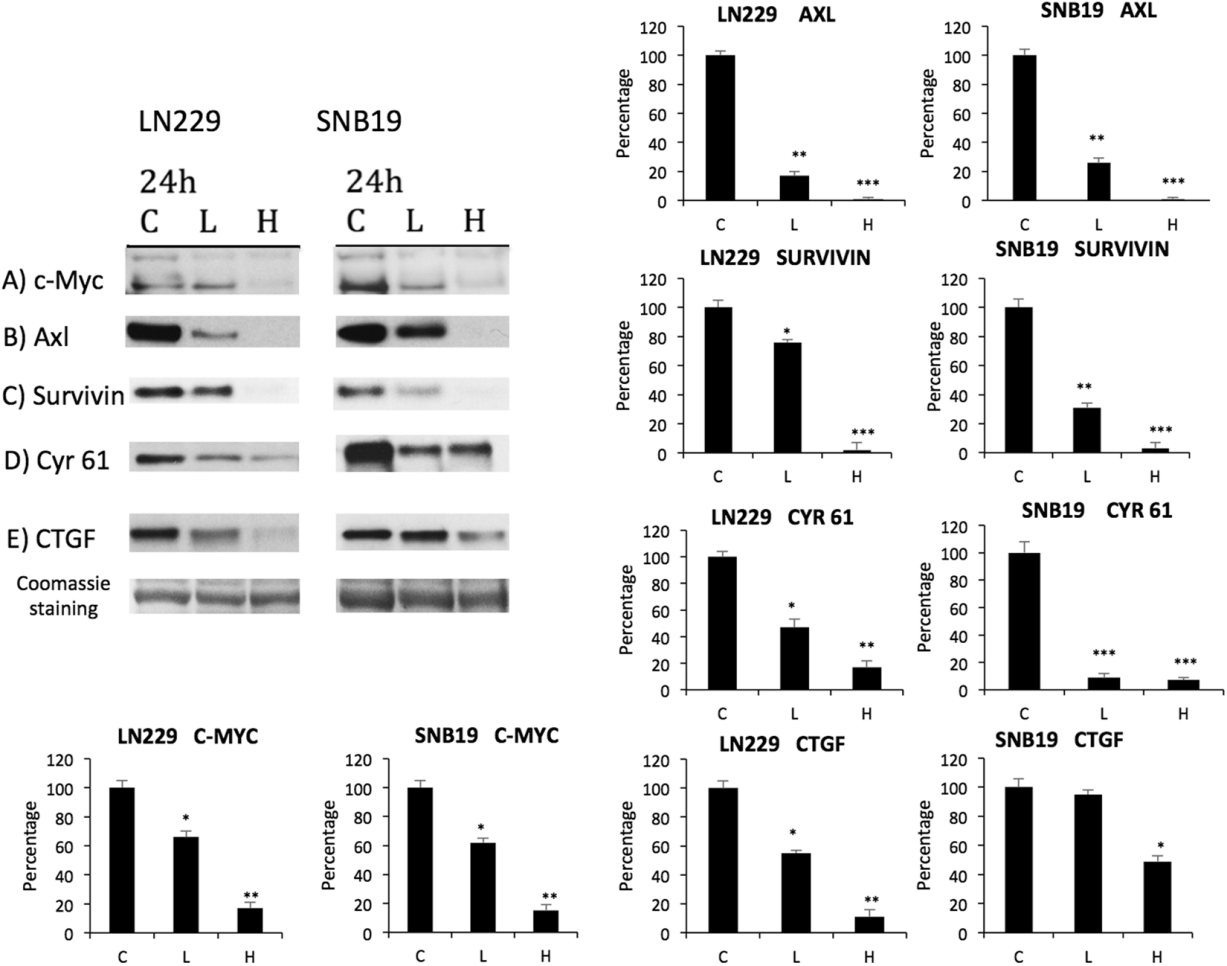
Verteporfin (VP) downregulates several members of the YAP-TEAD effectors in glioblastoma cells. SNB19 and LN229 cells were treated with vehicle (C), 2 µg/ml of VP (L) or 10 µg/ml of VP (H) for 24 hours protected from light at all steps. Western blot of protein extracts were probed for (**A**) c-myc, (**B**) axl, (**C**) survivin, (**D**) cyr-61, and (**E**) CTGF. Cropped blots are shown. Quantification of the blots relative to Coomassie blue staining, as a loading control, is on the right. (Experiment repeated 3 times, representative blot is shown here).

We next examined whether cells exposed to VP had a change in the expression of genes regulated by TEAD. At 24 hours, cells treated with VP (2 µg/ml and 10 µg/ml) demonstrated a dose-dependent downregulation of pro-proliferative molecules that are affected by a YAP-TEAD interaction, including c-myc, axl, survivin, cyr-61 and CTGF (Fig. 2A–E).

### VP down-regulates VEGFA expression and pluripotency marker Oct-4 in human glioma cells

**Figure 3:**
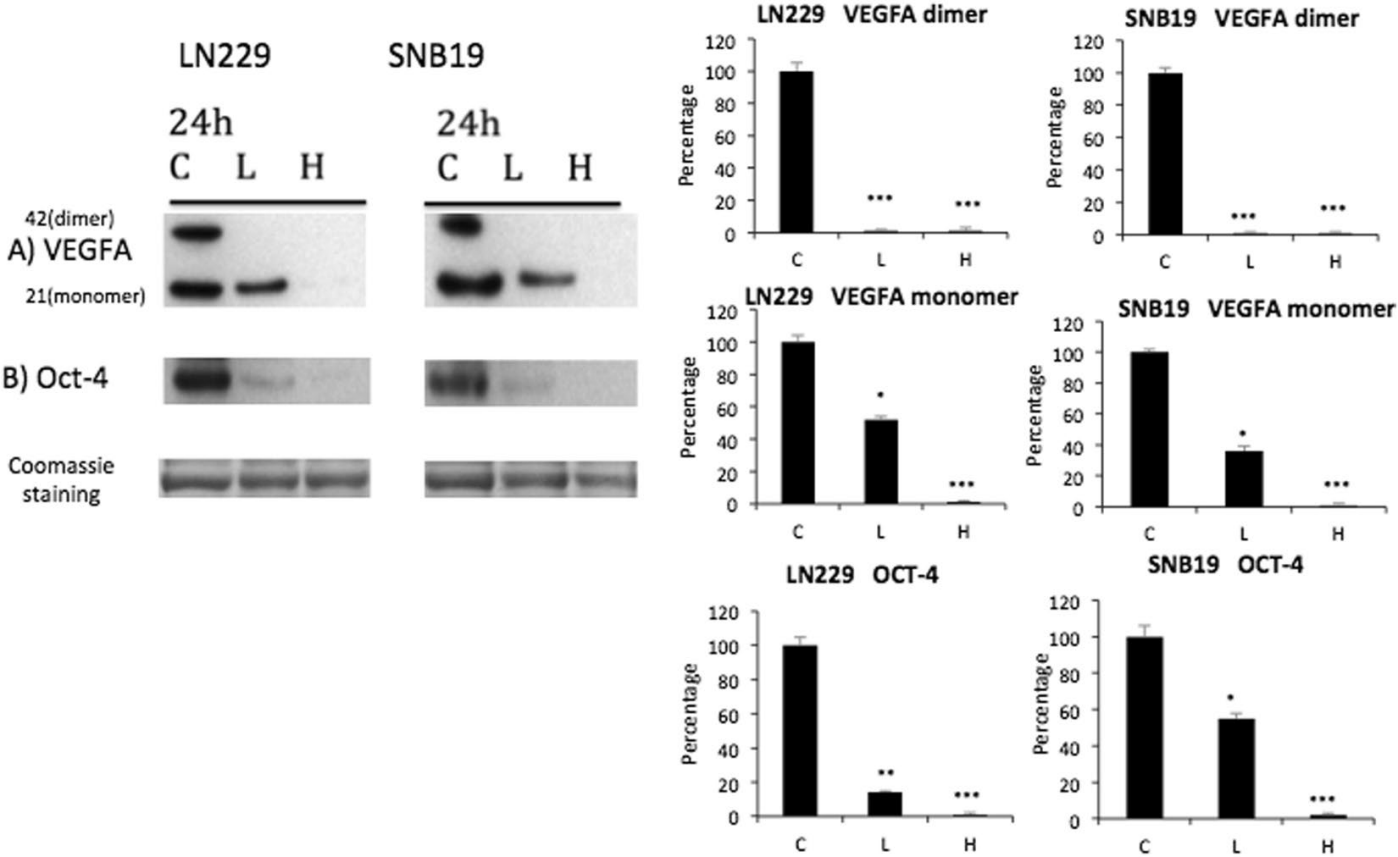
Verteporfin (VP) inhibits expressions of VEGFA and OCT-4 in glioblastoma cells. SNB19 and LN229 cells were treated with vehicle (C), 2 µg/ml of VP (L) or 10 µg/ml of VP (H) for 24 hours protected from light at all steps. Western blot of protein extracts were probed for (**A**) VEGFA, (**B**) pluripotency marker OCT-4. Cropped blots are shown. Quantification of the blots relative to Coomassie blue staining, as a loading control, is on the right. (Experiment repeated 3 times, representative blot is shown here).

It has been previously shown that TEAD is involved in VEGF, a key mediator of angiogenesis in cancer, expression via interaction with Vestigial-like (Vgll) transcription coactivators^34^. We demonstrated using western blot analyses that a dose-dependent decrease of VEGFA-expression was observed in glioma cells that were exposed to VP (Fig. 3A). OCT-4, a pluripotency cell marker that is expressed in cancer cells including cancer stem cell subpopulations, has been shown to be highly expressed in human gliomas and this expression increases in parallel with increasing glioma grades^35^. The YAP-TEAD pathway has also been shown to interact with OCT-4^36^. Our finding that VP causes a downstream decrease in YAP-TEAD pathway associated molecules prompted us to test the effect of VP on OCT-4 in glioma cells. Our results show that exposure of the glioma cells to VP is associated with a reduction in OCT 4 (Fig. 3B).

### VP activates p38 MAPK in human glioma cells

**Figure 4:**
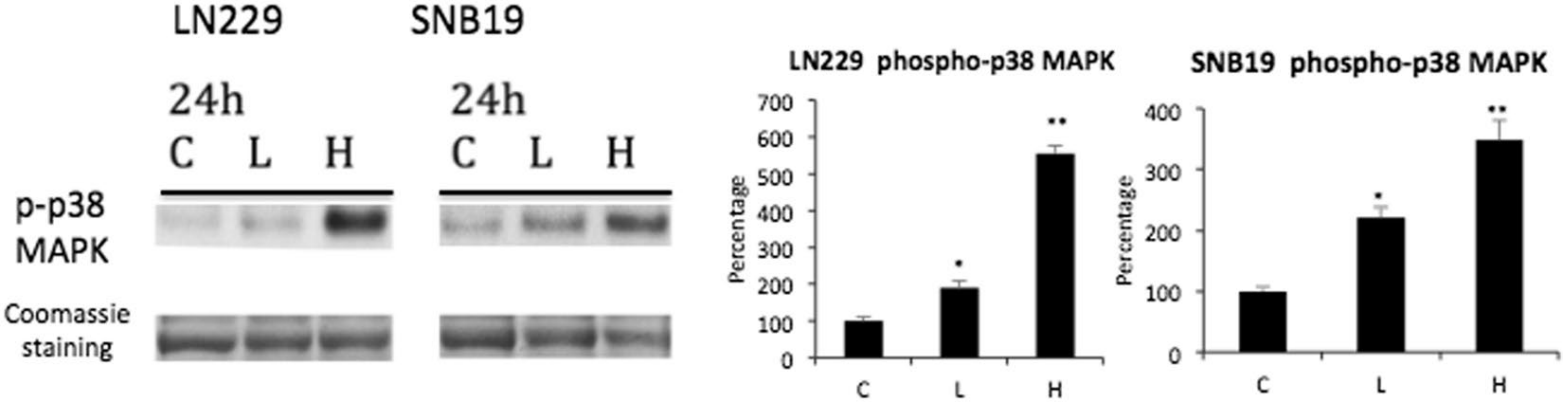
Verteporfin (VP) activates p38 MAPK glioblastoma cells. SNB19 and LN229 cells were treated with vehicle (C), 2 µg/ml of VP (L) or 10 µg/ml of VP (H) for 24 hours protected from light at all steps. Western blot of protein extracts showed that VP treatment induced phosphorylation of p38 MAPK in a dose-dependent manner. Cropped blots are shown. Quantification of the blots relative to Coomassie blue staining, as a loading control, is on the right. (Experiment repeated 3 times, representative blot is shown here).

A major regulator of the cell cycle and apoptosis are mitogen-activated protein kinases (MAPKs)^17^, and p38 MAPK activation is associated with an inhibition of tumor growth. In our study, exposure of glioblastoma cells to VP was also associated with induced expression of p38 MAPK (Fig. 4).

### VP does not affect the mTOR pathway while it slightly increases LC3-IIB protein levels in human glioma cells

**Figure 5:**
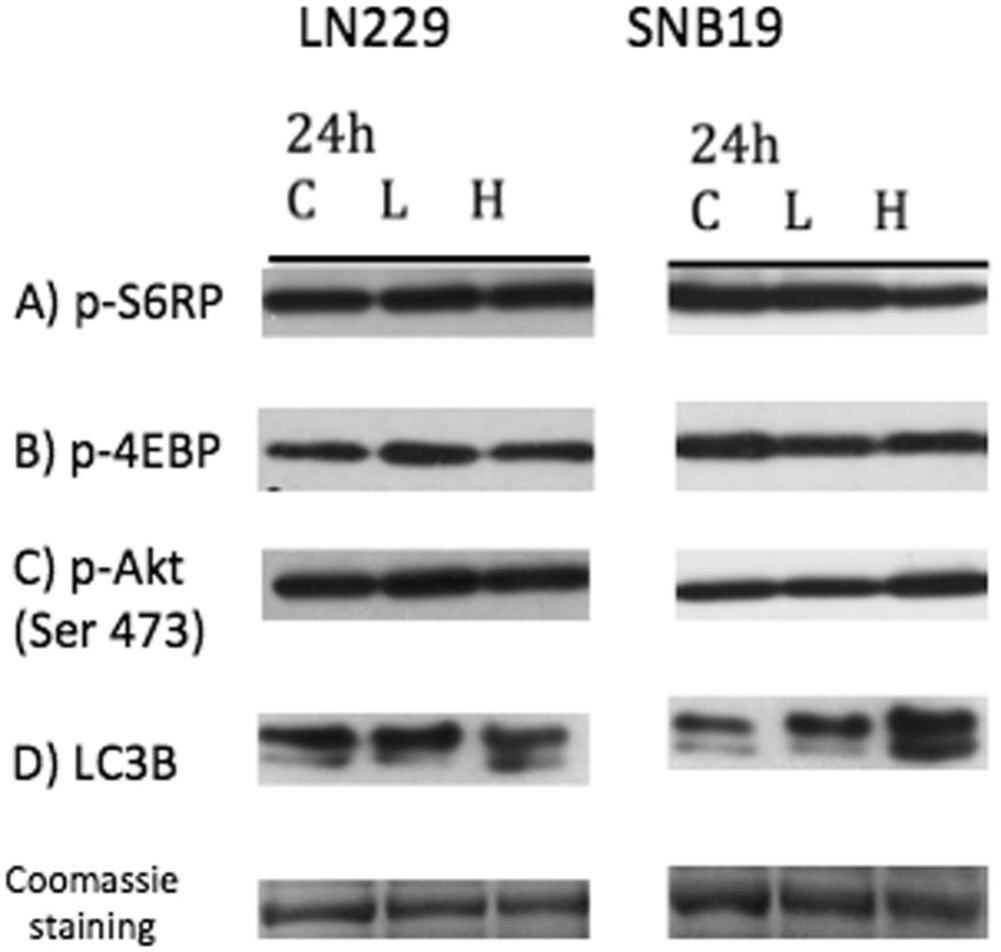
Verteporfin (VP) does not affect the mTOR or Akt pathway in glioblastoma cells, but increases LC3-IIB. SNB19 and LN229 cells were treated with vehicle (C), 2 µg/ml of VP (L) or 10 µg/ml of VP (H) for 24 hours protected from light at all steps. Western blots of whole cell lysates did not show an alteration in the phosphorylation status of ribosomal S6 protein (**A**) or of (**B**) p4EBP1 after VP treatment without light activation. (**C**) No changes in the phosphorylation levels of Akt (S473) were observed. (**D**) VP treatment of glioblastoma cells led to an increased expression of autophagic marker LC3-IIB in a dose-dependent manner. Cropped blots are shown. (Experiment repeated 3 times, representative blot is shown here).

Recently it was determined that YAP mediates crosstalk between the Hippo and PI(3)K–mTOR pathways and activates the mammalian target of rapamycin (mTOR), which is a major regulator of cell growth^19^. Western blots of whole cell lysates showed that VP treatment does not affect the phosphorylation status of ribosomal S6, p4EBP1 protein (Fig. 5A,B), or Akt Ser 473 (Fig. 5C). VP treatment led to a small increase in LC3-IIB protein levels in glioblastoma cells (Fig. 5D).

## Discussion

Despite the recent advances, malignant glioma remains a disease that results in death within a short period of time. In addition, in the pediatric population, treatment of benign glioma is associated with significant morbidity, underscoring the need for new targets and less toxic therapies. In this study, we demonstrate that human glioblastoma cell growth is inhibited *in vitro* when exposed to VP without the presence of light activation. VP may have a direct inhibitory effect on retinoblastoma, hepatocarcinoma, and ovarian cancer cells growth via disruption of the YAP-TEAD complex and the prevention of YAP-induced oncogenic growth^10, 30, 31^. YAP has been identified as a transcription coactivator that binds several transcription factors, including TEAD that is crucial for cellular development and cancer progression^29^. TEAD can also interact with multiple other coactivators that play an important role in cancer progression, such as Vgll proteins that upregulate the expression of VEGFA gene, the pro-angiogenic factor involved in cancer pathogenesis^34^. TEADs and their coactivators facilitate the progression of many cancers, including glioblastoma, through the upregulation of genes mediated by YAP, including c-myc, survivin, Axl, CTGF and Cyr61^37, 38^. The function of these genes is to promote cellular proliferation, tumor cell invasion, inhibition of apoptosis, metastasis, and epithelial-mesenchymal transition^39–42^. In our study VP treatment was associated with downregulation of these procancer proteins as well as downregulation of the proangiogenic protein VEGF.

In addition to VP’s effects on pathways that promote cancer growth, treatment of glioblastoma cells with VP was also associated with an increased expression of LC3IIB, a marker of autophagosome biogenesis. Accumulation of autophagosome may represent either autophagy induction or, alternatively, suppression of the autophagy pathway downstream of autophagosome formation^43^. In fact, Donohue E *et al*. showed that VP is a late stage autophagy inhibitor in MCF-7 cells^44^. Autophagy, the process of degrading unnecessary cellular proteins and organelles through the actions of lysosomes, can be an appropriate response to stress and promote survival, but persistent stress can lead to autophagic (or programmed type II) cell death^45^. Therefore, the increased expression of LC3IIB suggests that verteporfin may affect cancer autophagic cell death, but this effect could be different among different cancer types and different experimental conditions. VP has been shown to induce, in a non-light dependent mechanism, the formation of cross-linked oligomers and high molecular weight protein complexes (HMWC) that could interfere with autophagy and cancer cell growth^46, 47^. More recently, our group showed that the prevalence of at least some HMWCs is likely due to the presence of ambient light during post-lysis analysis^48^. Our data in the current study suggests that the observed downregulation of some YAP-TEAD-associated downstream signaling molecules in darkness is not due to HMWC formation (Supplementary Figures 1 and 2) and that VP may have direct effects on some cancer growth pathways.

An anti-proliferative effect of verteporfin on human glioblastoma cells was also observed through activation of p38 MAPK, which has been implicated in the inhibition of tumor growth^49^. Activation of p38 MAPK induces apoptosis in multiple cancer cell lines, including acute lymphoblastic leukemia and hepatoma cells^50^, and also promotes the mitochondrial apoptotic pathway^51^. We therefore suggest that p38 may mediate VP’s effect on glioblastoma cells. Interestingly, VP treatment of human glioblastoma cells did not affect Akt or mTOR pathway, which is in contrast to our prior results on the effect of VP on human retinoblastoma^31^. This is a surprising finding because YAP mediates crosstalk between the Hippo and phosphatidylinositol 3-kinase (PI3K)/AKT signaling pathways and it activates the mammalian target of rapamycin (mTOR), signaling pathways that are important for the promotion of tumor proliferation, migration, and apoptosis^40–42, 49^.

Additionally in this study we found that verteporfin downregulated the pluripotent marker Oct-4, a transcription factor that is expressed in cancer stem cells and in human glioma cells^35^. This builds on our prior work that suggests that targeting OCT-4 may beneficial in treating some cancers and supports suggested role of YAP/TEAD signaling pathway in pluripotency^31^.

In summary, we demonstrate for the first time that verteporfin can inhibit growth and proliferation of human glioma cells without any light activation *in vitro* and that this inhibition of cell growth correlates with downregulation of some YAP/TEAD-associated downstream signaling molecules. It also shows for the second time that Verteporfin may interfere with pluripotency marker OCT-4. Our study supports the role of VP as a potential therapy for multiple solid tumors, such as retinoblastoma, hepatocarcinoma, ovarian cancer, and glioma. Further studies are needed to elucidate the exact mechanism of VP action and supports further exploration in animal research.

## Electronic supplementary material

Supplementary figure
