# Supplementary figures and images for: Verteporfin inhibits growth of human glioma in vitro without light activation
Source: Sci Rep. 2017 Aug 8;7:7602. doi: 10.1038/s41598-017-07632-8 (PMC5548915; doi:10.1038/s41598-017-07632-8)

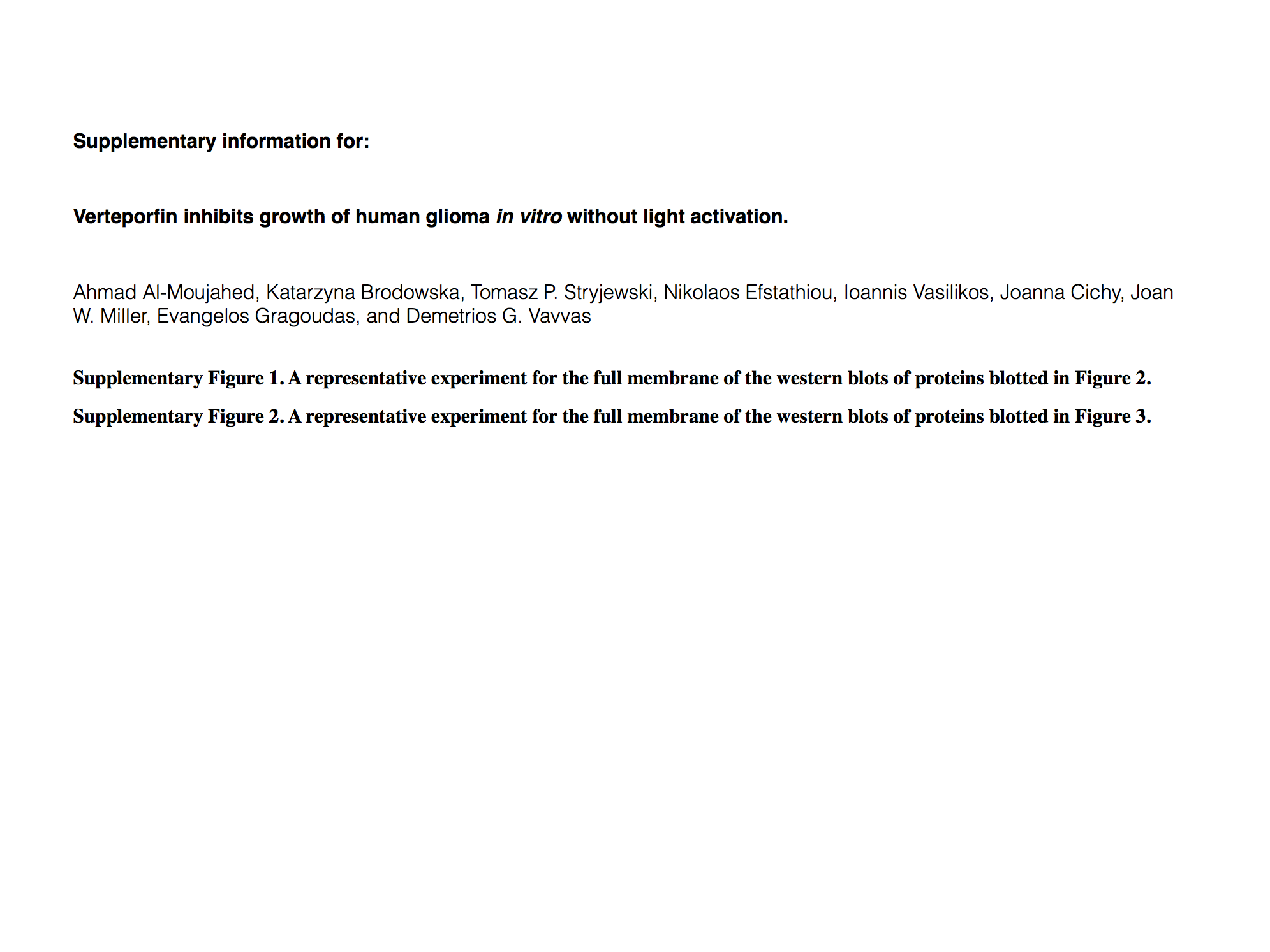

Supplement: Supplementary file 1 — Supplementary figure [file 41598_2017_7632_MOESM1_ESM.tif]
